# Which Topical Microbicides for Blocking HIV-1 Transmission Will Work in the Real World?

**DOI:** 10.1371/journal.pmed.0030351

**Published:** 2006-08-15

**Authors:** Per Johan Klasse, Robin J Shattock, John P Moore

## Abstract

Klasse and colleagues discuss key research on the four crucial issues in developing a microbicide: safety, acceptability, efficacy, and affordability.

A topical microbicide can be used vaginally or rectally by a woman or a man to reduce the risk of acquiring HIV-1 infection during sexual intercourse [1–4]. An effective microbicide could make a significant contribution to reducing the global spread of HIV-1. What will it take to make one? What types of inhibitory compounds could be developed as a practical product, and what obstacles must be faced? In this article, our emphasis will be on newer technologies (Table 1, “Specific Entry Inhibitors”), rather than the earlier-generation surfactants, pH-modifiers, and polyanions (Table 1, “Non-Specific Entry Inhibitors”) that are already being tested in large-scale clinical trials [5] and have been reviewed extensively elsewhere [1,2,4].

**Table 1:**
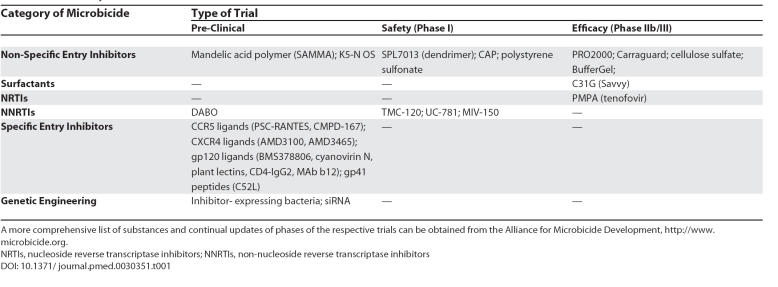
Summary of Pre-Clinical and Clinical Trials of Microbicides

We believe a successful microbicide would have to fulfill four interrelated criteria: safety, acceptability, efficacy, and affordability. We will address each issue in turn, discussing how it might affect the development of various microbicide concepts.

## Safety

Safety will, of course, be of paramount importance. The microbicide field suffered a significant setback when efficacy trials of the surfactant nonoxynol-9 showed that the HIV-1 transmission rate was greater in the active group than in the placebo [6]. The most likely reason was that, in sustained repetitive use, nonoxynol-9 disrupted the vaginal epithelium, damaging an important natural barrier against HIV-1 [6]. Hence any practical microbicide will need to preserve the body's natural defenses. An example of such a defense is the film of lactobacilli that maintains a low vaginal pH, which lowers the risk of HIV-1 infection. In contrast, vaginosis caused by anaerobic bacteria raises the pH and the risk of HIV-1 infection [7].

For any substance that is repeatedly applied to a mucosal site, carcinogenicity and teratogenicity must also be carefully evaluated over the long term. It is not sufficient merely to assess the effects of microbicides on the lower genital tract of healthy women; safety studies must also involve women with genital ulceration and inflammation caused by other sexually transmitted diseases, or with cervical ectopy (outgrowth of the delicate cervical columnar epithelium onto the ectocervix, particularly common in young women). It is also highly likely that microbicides will enter the uterus, which is very susceptible to toxicity effects [8,9]. Microbicides are likely to be used rectally by men and women, and the rectal tract environment is particularly sensitive to agents that disrupt cell membranes or mucosal barriers [10–12].

Despite the widely recognized problems with nonoxynol-9, efficacy trials of another surfactant-based microbicide, C31G vaginal gel (Savvy) are in progress. Some Savvy components can damage or inflame epithelial tissues, particularly in the cervix [13]. Another high-profile failure on safety grounds could have very serious consequences for the long-term future of the microbicide concept.

If microbicides are used in the same populations targeted by vaccine trials, it will be important to determine whether they affect localized immune responses to vaccines. Conversely, microbicides should not cause localized inflammation, because attracting immune system cells to the sites of virus deposition would probably facilitate transmission [14,15]. Compounds that could be immunogenic, such as peptides and proteins, or compounds that could affect the normal trafficking of immune system cells, such as chemokine derivatives, will need to be studied carefully from this perspective. CpG oligonucleotides and imiquimod, both activators of innate immunity via Toll-like receptors, did not protect against vaginal transmission of simian immunodeficiency virus to macaques and may even have exacerbated it by causing local inflammation [16]. Mucosal biopsies during Phase I trials can help determine whether candidate microbicides are inflammatory in humans.

Women who are already infected with HIV without knowing it are certain to use any licensed microbicide product. Hence, the development of drug resistance is also a safety concern, particularly for reverse transcriptase or entry inhibitors similar to ones used to treat HIV-1 infection [17–19]. It seems unlikely that topically applied inhibitors would generate systemic concentrations sufficient to exert a significant selection pressure [19,20]. However, local resistance could be fostered within mucosal sites close to the site of application, perhaps leading eventually to the systemic emergence of resistant variants. Microbicide usage in a geographic area where strains resistant to that drug class are already present may also constitute a selective pressure, facilitating transmission of resistant viruses. Clinical studies will need to address this issue, analogous to when antiviral drugs are used to prevent mother-to-child transmission [21].

A related point is whether a vaginally applied inhibitor specific for CCR5-using (R5) viruses could promote, as opposed to passively permit, the transmission of CXCR4-using (X4) variants that are associated with more rapid disease progression [2,19,22,23]. X4 virus transmission is rare, and any increase would be a cause for concern [15,23,24]. One solution would be to combine a CCR5 inhibitor with other entry or post-entry inhibitors that are active against X4 viruses.

## Acceptability

Acceptability is a real-world concept rarely considered by academic scientists working on microbicide development in vitro or with animal models. It is, however, a critical issue for a final product that is to be used in a sexual setting. Sex is often spontaneous, conducted in the dark, and can be associated with alcohol and/or drug use. If a microbicide is difficult to use, has the wrong consistency (too viscous or too fluid), has an unusual smell or color, or just comes across to the users or their sexual partners as being “nasty” or culturally unacceptable, then it will simply be rejected. Early consideration should be given to such points.

The duration of protection that can be achieved by a topically applied compound may affect its acceptability. Ideally, it should not be necessary to apply a microbicide only moments prior to intercourse; an efficacy window measured in hours or longer will be an important feature. Intra-vaginal devices that release active compounds slowly over sustained periods are under evaluation [25]. Even so, it is advisable for microbicide developers to assess the longevity of protection by performing delayed challenge experiments in animal models.

When small-molecule entry inhibitors were tested in the rhesus macaque vaginal challenge model, significant protection was achieved even with a six-hour delay before challenge [19]. The vaginal application of lipid-formulated small inhibitory RNA (siRNA) to herpes simplex virus (HSV) structural genes protected mice against a lethal vaginal challenge with HSV a few hours later; in other studies, non-viral target genes remained silenced for at least nine days, raising the possibility of achieving truly sustained protection against HIV-1 and other viruses [26].

Acceptability issues also apply to genetically modified commensal bacteria, such as lactobacilli or *E. coli*, that are altered to secrete or express proteins with anti–HIV-1 activity [27–29]. The transgene product could be immunogenic, or it might change the pathogenicity of the engineered organism. The real risks of this approach are probably very low, but problems with political or public perception may arise: genetically modified food has attracted considerable controversy worldwide. It might not be easy to persuade a nation such as Zambia, which has rejected genetically modified corn shipments paid for by Western donors, now to permit widespread vaginal and rectal colonization by similarly engineered microorganisms made in American laboratories. In principle, similar objections could be raised to the use of siRNA as a microbicide if the method is labeled as gene therapy rather than what it actually is: the topical application of a replication inhibitor.

## Efficacy

Efficacy is self-evident: a microbicide must work for it to be useful. One obvious reason for failure would be inconsistent use, either because it is not an acceptable product (see above) or because it is too expensive for those who need it most (see below). Adverse effects on epithelial integrity and immune activation could also impair efficacy (see above). However, a regularly used product might fail because it simply lacks the potency to protect against the HIV-1 strains it encounters. Thus, it will be essential to accurately identify the underlying causes of failure for any microbicide that is ineffective in Phase III trials; different problems will require different solutions.

But how can we identify what concepts are worth evaluating in efficacy trials? Not every candidate microbicide can be tested in Phase III trials, which are costly in terms of both volunteer numbers (2,000 to 12,000 participants) and money ($40 million to $70 million). There are also issues of public perception: multiple, sequential failures may compromise the prospects for future trials of more promising concepts. Four broadly similar polyanions (PRO2000, Carraguard, BufferGel, and cellulose sulfate), one surfactant (C31G), and one nucleoside reverse transcription inhibitor (PMPA) are now in Phase IIb/III efficacy trials, conducted by several different funding agencies (see Table 1 and http://www.microbicide.org). The success of any of these products would greatly boost the microbicide concept, but would also complicate the future testing of potentially more potent substances. To prove that a new candidate is more effective than a partially active first-generation microbicide would require a larger trial than a simple comparison with a placebo.

### Macaque models

The most practical way, albeit an imperfect one, to judge which microbicides would have the potential to be protective in humans is to use the macaque models. Many different compounds have now been shown to be able to protect macaques from vaginal and/or rectal challenge by a simian/human immunodeficiency virus (SHIV) or simian immunodeficiency virus [2–5]. We will consider the varied implications of these results, while noting that the onus should now be on all future potential microbicides to do at least as well as compounds that are effective in the macaque. Not every compound that is protective in the macaque will be useful in humans (e.g., nonoxynol-9) because the macaque model cannot mimic what happens in long-term use, and it can also be argued that a reliance on the macaque model could preclude potentially useful compounds from ever being evaluated in humans. But is it prudent to prioritize concepts that have not demonstrated the potential for efficacy above others that have, when hard and expensive choices must be made?

Using monkey models in this way will require reaching a consensus about which model(s) to use and what the results mean from a quantitative perspective. Only a few different challenge viruses are in widespread use, perhaps only a single R5 SHIV and two X4 SHIVs. One or two RT-SHIVs (expressing the HIV-1 reverse transcriptase) are also being evaluated. It should therefore be possible to agree on uniform challenge stocks.

### “Low-dose” versus “high-dose” challenge models

**Figure 1:**
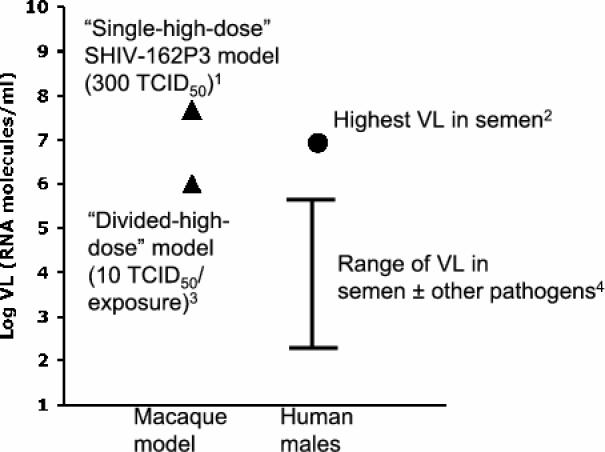
The HIV/SHIV Viral Load in Two Different Transmission Situations: Human Semen and the Inoculum in the Macaque Model For a detailed explanation of Figure 1, see Text S1.

More difficult, but surely not insuperable, is the issue of the “low-dose” versus “high-dose” challenge models (Figure 1), both of which are in use [19,22,30–36]. In fact, both models use challenge doses that are high compared with the infectious inoculum encountered by humans. Better terms for the two models might be “single-high-dose” and “divided-high-dose” since the cumulative amount of infectious virus is similar in each case, as is the cumulative animal infectious dose, which may be the most crucial parameter (Figure 1). Both models could be pursued, particularly if head-to-head comparison studies are performed involving the same test compounds and challenge viruses. The protection provided by the same concentration of the virus-binding entry inhibitor, CAP, seems very similar in the two models [32,36]. If both models provide much the same answer, as we suspect will be the outcome, the one to favor would presumably be the logistically simpler “single-high-dose” model.

One issue arising from animal model research relates to the inhibitor dose that would be required for prevention of HIV-1 transmission to a human. As the amount of compound required has a significant bearing on its cost (see below), and conceivably on its formulation (see above), this issue requires careful consideration.

**Figure 2:**
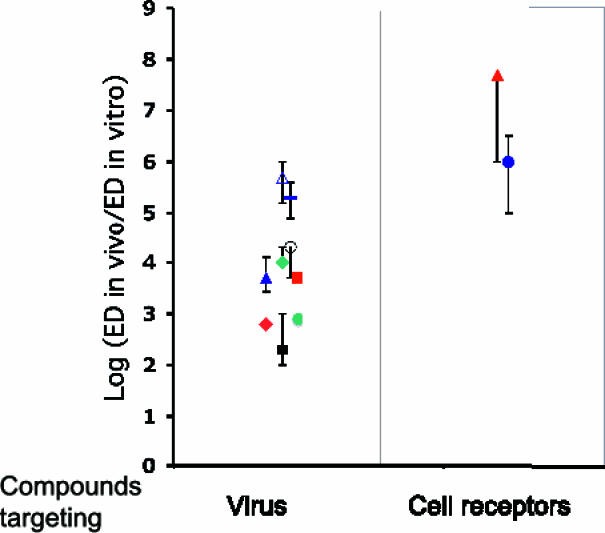
Comparison of the Concentrations of Microbicide Compounds Required for Blocking Infection In Vitro and In Vivo For a detailed explanation of Figure 2, see Text S2.

It has been generally found from studies in the “single-high-dose” macaque model that protection against vaginal or rectal transmission, irrespective of the challenge virus, requires the use of inhibitor concentrations in the millimolar range, 10^2^- to 10^7^-fold greater than doses effective in cell culture systems (Figure 2). Although only two receptor blockers, both targeted at CCR5, have yet been tested in the macaque, the available data suggest that the differential may be greater for compounds that target the cell than for those directed at the incoming virus (Figure 2). One reason could be that more inhibitor is needed to diffuse into mucosal tissues and occupy the available receptors than to encounter the virus in the fluid phase of the vaginal lumen. However, even for the virus-binding inhibitors, the ratio of the concentrations active in vivo and in vitro is still substantial (10^2^- to 10^6^-fold; Figure 2). Whether similar ratios will be observed with the “divided-high-dose” challenge model remains to be determined; we believe there will be little difference between the two models in this regard.

**Five Key Papers in the Field**

**Hillier et al., 2005** [6] A review of the epithelial damage caused by the early microbicide candidate nonoxynol-9 and how this enhances the risk of HIV-1 transmission.

**Lederman et al., 2004** [22] A modified form of a chemokine, PSC-RANTES, applied vaginally, was shown to protect macaque monkeys from challenge with a chimeric SHIV.

**Palliser et al., 2006** [26] This paper shows how siRNA molecules targeting viral genes, when applied vaginally in a murine model, can protect against lethal infection with HSV type 2.

**Stone, 2002** [1] This review introduces the concept of vaginal and rectal microbicides and gives a historical perspective on the development of the field. It describes different classes of drugs that might make suitable microbicides.

**Veazey et al., 2005** [19] Different classes of entry inhibitors, small-molecule CCR5, and Env ligands, alone and in combination, were shown to protect macaque monkeys from challenge with a chimeric SHIV.

Differences as large as those noted in Figure 2 cannot be attributed to the use of much higher amounts of virus in vivo. In some studies, the tissue culture infectious dose (TCID_50_) used for in vitro infection assays and for in vivo challenge was identical [19,22]; in another, only 3-fold more virus was used in vivo than in vitro [36]; and the amount of virus applied in an in vitro assay using cervical tissue explants was actually 6-fold *higher* than the amount that was used to challenge macaques in the same study [19]. Furthermore, since the volumes used in vitro are smaller than those used for delivery of an inoculum in vivo, the virus concentrations are actually higher in vitro. In only one study was substantially (100- to 1,000-fold) more virus applied vaginally than was used in tissue culture assays [37].

Also, in any given in vitro assay, an increase in viral dose does not mean a proportional increase in the amount of inhibitor required to counter it. Indeed, there is normally a zone of viral input over which the effective inhibitor concentration (present in great molar excess over its target) is approximately constant. The “Percentage Law” covers this scenario [38]. By comparing the fractions of animals infected after receiving low and high doses of virus in the two models, we can also conclude that the infectivity of the inoculum does not have to be reduced more than about an order of magnitude to achieve partial protection [30–32,35]. Hence the requirement for high inhibitor concentrations for protection of macaques from vaginal challenge is unlikely to have a virological basis; it more probably reflects the pharmacology of inhibitor delivery to sites where virus is deposited and/or where it first encounters its cell surface receptors.

The above discussion has implications for the critical question: Will much less inhibitor be required to protect a woman from an infectious man than a macaque from a researcher with a syringe full of cell-free virus? We believe it would be imprudent to make this assumption. A comparison of the limited datasets available to date from the “single-high-dose” and “divided-high-dose” models shows that a proportional relationship between the efficient dose of blocker and the infectious dose of virus cannot be assumed. Such models do not account for the effects of sexual intercourse, the length of time between application and exposure to infectious virus, and the potential diluting effects of vaginal fluid and semen. Furthermore, these models have yet to be adapted to evaluate potential inhibitors of cell-mediated transmission by the virus-containing lymphocytes present within an infectious ejaculate.

### Using high concentrations of multiple inhibitors

Taking all the above factors into account, we believe the strategy most likely to be effective in women would be to use high (millimolar range) concentrations of more than one inhibitor. Using combinations has obvious advantages for prevention, just as it does for treatment of established infection.

For example, combinations could reduce the transmission of viruses insensitive to one or more components, provide additive or synergistic potency (allowing dose-sparing), and increase the breadth of coverage against the divergent strains that a microbicide must combat in the real world. However, there may be practical obstacles to combinations: at present, individual compounds must first be approved separately, and it will be a complex process to optimally co-formulate different substances, then to prove in clinical trials that all the constituents are active. Also, interactions between inhibitors may cause unexpected toxicities.

A candidate microbicide's breadth of reactivity against circulating HIV-1 strains cannot, of course, be evaluated in the monkey model, because only a few challenge viruses are available. However, panels of suitable test viruses from multiple genetic subtypes have been assembled for in vitro evaluation of vaccine-induced neutralizing antibodies [39–41]. The genetic variation issues that bear on preventing HIV-1 transmission are similar for microbicides and for vaccine-induced neutralizing antibodies; the vaccine test panels should serve a dual purpose.

### Microbicidal protection by engineered commensal bacteria

**Figure 3:**
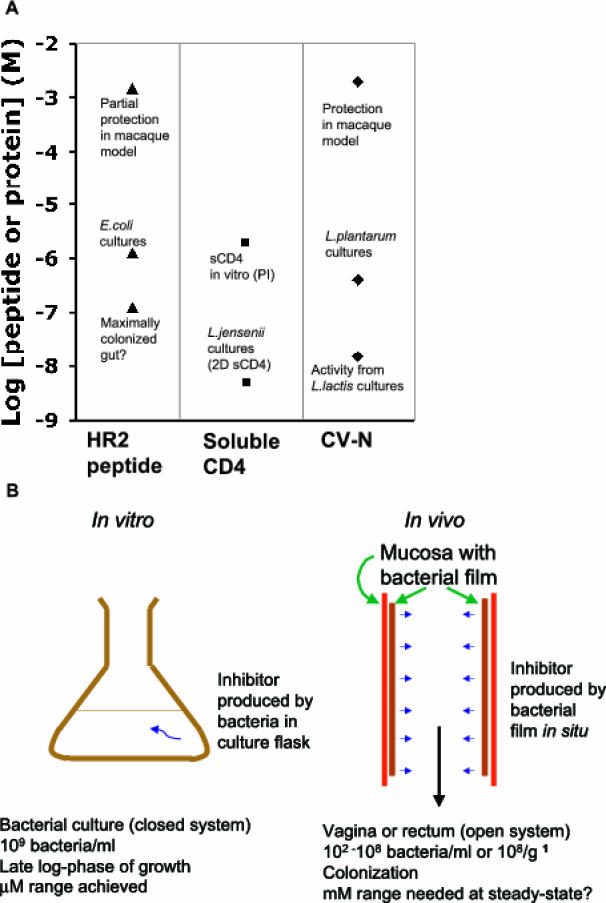
Microbicidal Protection by Engineered Commensal Bacteria For a detailed explanation of Figure 3A, see Text S3. For a detailed explanation of Figure 3B, see Text S4.

One sophisticated approach to achieving the continuous presence of a viral blocker in mucosal tissues is colonization of the vagina or rectum with recombinant bacteria secreting fusion inhibitory peptides or proteins [27–29]. Some quantitative aspects of this strategy are illustrated in Figure 3. Firstly, the inhibitor concentrations secreted in bacterial cultures are sometimes not sufficient to block infection by primary isolates. Secondly, there is a large gap between in vitro–inhibitory and in vivo–protective concentrations (see Figure 2). Thirdly, lactobacilli in the vagina occur naturally in thin films at varying but lower densities than in bacterial cultures. Fourthly, the continuous losses and dilution of substance secreted into the vaginal or rectal lumen will reduce the cumulative inhibitor concentration. Whether the engineered-lactobacilli approach to a microbicide could be effective will depend on whether inhibitor concentrations present at steady state in a bacterial film are sufficient to counter the incoming virus. The concept now needs to be evaluated in the macaque model, to determine the inhibitor concentrations present in vivo and whether they can protect against a SHIV challenge.

## Affordability

Affordability is relevant because a microbicide must be manufactured at a cost that allows the product to be either given away or sold at a price accessible to developing-world users. Funding by governments or international agencies will affect what price is realistic. However, it would be hard to make the case that a microbicide product could cost much more than $1 per application; ideally it would be much less. Some sophisticated, high-tech approaches may represent outstanding science, but would simply be too expensive to apply.

We will address this point by considering the economics of developing the C52L peptide as a microbicide product. C52L is a bacterially expressed, sequence-modified version of the licensed drug enfuvirtide (T-20). We showed it to be broadly inhibitory against diverse HIV-1 strains in vitro, to act synergistically with other entry inhibitors (CMPD 167 and BMS-378806), and to protect macaques from vaginal transmission of SHIV-162P3, both alone and in combination with other entry inhibitors [19]. Such properties suggest it could make a valuable contribution to a multi-component, entry inhibitor–based microbicide. However, although the International Partnership for Microbicides has reached agreements with Merck and Bristol-Myers Squibb to develop CMPD 167 and BMS-378806 as a practical microbicide product(s), C52L was not the subject of a similar agreement [42]. The reason is its likely high cost of manufacture on a large scale, particularly to the good manufacturing practice requirements necessary for human trials.

Broadly similar considerations may apply to developing other peptides or proteins such as PSC-RANTES and cyanovirin-N that have also been protective in monkey models [22,34,35]. PSC-RANTES is about a log more potent than C52L in vitro and in vivo, but because chemical modifications to the basic chemokine structure are required, it is more difficult to make [3]; cyanovirin-N may also be hard to express in large quantities.

Economies of scale are possible for any approved product that must be made in bulk, and cost-efficiency improvements might be feasible (e.g., the development of fermentation technologies, plant-based expression systems, and new chemokine derivatives that can be expressed efficiently in bacteria). Significant investments must now be made to cover the start-up costs associated with the development of such potentially more economic methods to produce protein- and peptide-based microbicides.

The cost of making enough siRNA to protect a woman from vaginal HIV-1 transmission has been estimated to be about $8 per dose, based on what was needed to protect mice from lethal HSV infection [26]. This is perhaps an order of magnitude too expensive for routine use. However, if siRNA can provide durable protection, as suggested by the longevity of gene silencing in mice, it might be possible to apply it infrequently, perhaps weekly, reducing the cost to acceptable levels.

A non-nucleoside reverse transcriptase inhibitor provides prolonged protection in tissue explant experiments [43]. The sustained release of such an inhibitor from silicone elastomer rings, designed for intravaginal application, can maintain concentrations in vitro well above those required to prevent explant infection [25]. While vaginal rings may cost about $5 to $10, they can be loaded with enough drugs to last several months. Clearly, compounds and delivery methods that provide prolonged protection may reduce costs while improving both efficacy and acceptability.

## Conclusion

Not every scientifically sound concept can be developed as a product for use in the real world. Every concept has its challenges; while some will be overcome, other obstacles may be insuperable. Our intention has been to highlight some of the more obvious issues that affect all, or some, of the many promising microbicide concepts now being considered for product development.

## Supporting Information

Text S1 Detailed Explanation of Figure 1
(67 KB DOC).

Text S2 Detailed Explanation of Figure 2
(69 KB DOC).

Text S3 Detailed Explanation of Figure 3A
(58 KB DOC).

Text S4 Detailed Explanation of Figure 3B
(64 KB DOC).

## Abbreviations

HSV: herpes simplex virus
SHIV: simian/human immunodeficiency virus
siRNA: small inhibitory RNA
