# Supplementary material for: Which Topical Microbicides for Blocking HIV-1 Transmission Will Work in the Real World?
Source: PLoS Med. 2006 Aug 15;3(9):e351. doi: 10.1371/journal.pmed.0030351 (PMC1539095; doi:10.1371/journal.pmed.0030351)
Supplement: Supplementary file 1 [file pmed.0030351.sd001.doc]

Text S1. Explanation of notes 1–4 in figure 1.

The HIV/SHIV inoculum (log RNA copies) per ml in different transmission situations is depicted on the y-axis.

^1^In the “single high-dose” model of vaginal R5 virus transmission to progesterone-treated macaques, an infectious dose of 300 TCID_50_ of SHIV-162P3 was used, which corresponds to >2 monkey infectious units (MIU) [19]. In a “single high-dose” model of vaginal X4 virus transmission, the *in vitro* infectious dose of SHIV-89.6PD (2 x 10^4^ TCID_50_) was higher, but it corresponds to a lower apparent monkey infectious dose (~ 1.4 MIU) because the monkeys were not progesterone-treated [37]. We cannot extrapolate the RNA copy number from the TCID_50_ for this particular stock, so the viral dose is not plotted. Intrarectal inoculation with 1000 TCID_50_ of SHIV-89.6P or intravaginal inoculation, after progesterone treatment, with 5000 TCID_50_ of SHIV89.6P led to infection of all the control animals, but again the RNA copy number cannot be extrapolated [34,35].

^2^The highest HIV-1 RNA copy number measured in semen of infected patients [44] cannot be translated into a TCID_50_ value: attempts to isolate HIV-1 from semen are generally unsuccessful. However, the highest risk of transmission from man to woman per coital act is estimated to be 10%, which is likely to reflect the coincidence of the highest infectiousness of the transmitter with the highest susceptibility of the recipient [14]. This would correspond to ~ 0.1 human infectious unit.

^3^The copy number of SHIV-162P3 in the “divided high-dose” model of vaginal transmission to macaques [32] has been extrapolated from the TCID_50_. The value plotted corresponds to the individual dose of one exposure. The cumulative dose required to infect control animals can be as low as 30 TCID_50_ but the challenges continue to > 100 TCID_50_ which is close to that in the “single high-dose” model. At identical concentrations of the microbicide CAP, a similar degree of protection was achieved in the two models (cf. Figure 2). In another “divided high-dose” model, of intrarectal SIVmac239 inoculation, with 30 TCID_50_ per dose, on average 8 doses were required for infection of controls, with 300 TCID_50_ per dose, on average 5.5 doses were required and with a dose of 3000 TCID_50_ a single dose was sufficient [31].

^4^The approximate range of mean VL in human semen from HIV-1-infected men is given for patients who are also infected with other pathogens causing urethritis (highest), and for those without other identified pathogens (lowest; often undetectable). The VL in semen is higher in primary infection [45] and in the absence of ART [7]. Partly because of these factors, it is also subject to geographical variation [46].
