# Supplementary material for: Which Topical Microbicides for Blocking HIV-1 Transmission Will Work in the Real World?
Source: PLoS Med. 2006 Aug 15;3(9):e351. doi: 10.1371/journal.pmed.0030351 (PMC1539095; doi:10.1371/journal.pmed.0030351)
Supplement: Supplementary file 2 [file pmed.0030351.sd002.doc]

Text S2. Detailed explanation of figure 2.

On the y-axis is plotted the log of the ratio of the *in vivo* over the *in vitro* concentrations that gave equivalent, usually ~50%, protection or block of replication (in macaque PBMC, when such data are available). Substances that bind to the virus before or after encounters with target cells are shown to the left, those that bind to the target cells (the CCR5 coreceptor) are on the right. Left-hand column: SPL7013 against X4 virus [47] (blue filled triangle); CAP against X4 virus [36] (red diamond); CAP against R5 virus [36] (red square); CV-N against X4 virus in rectal application [35] (green diamond) or in vaginal application [34] (open circle); PRO-2000 against X4 virus [37] (green circle); b12 against R5 virus [48] (black square); BMS-378806 against R5 virus [19] (blue open triangle); C52L against R5 virus [19] (blue bar). Right-hand column: CMPD167 against R5 virus [19] (red triangle); PSC-RANTES against R5 virus [22] (blue circle). Error bars indicate the range of ratios based on variation in effective concentrations within assays or in different assays *in vitro,* or uncertainty about the concentration dependence of partial protection *in vivo*.
