# Supplementary material for: Which Topical Microbicides for Blocking HIV-1 Transmission Will Work in the Real World?
Source: PLoS Med. 2006 Aug 15;3(9):e351. doi: 10.1371/journal.pmed.0030351 (PMC1539095; doi:10.1371/journal.pmed.0030351)
Supplement: Supplementary file 3 [file pmed.0030351.sd003.doc]

Text S3. Detailed explanation of figure 3A.

The plot illustrates the quantitative problem of blocking HIV-1/SHIV infection by colonizing the vagina or rectum with a commensal recombinant *E.coli* strain that secretes gp41-derived (HR2) fusion-blocking peptides (left), or with lactobacilli secreting a 2-domain CD4 molecule (middle), or with lactobacilli secreting Cyanovirin-N (CV-N) (right). On the y-axis, the log concentrations (M) of the inhibitors are given. The concentration of a similar HR2 peptide (C52L) supplied vaginally in purified form, which achieved only partial protection of macaques [19], is ~3 logs higher than the concentration of secreted peptide in a culture of recombinant *E.coli* in exponential growth phase [29] (left column). Middle column: A two-domain CD4 construct was expressed in commensal lactobacilli *in vitro*. The accumulated protein concentration in the bacterial culture, ~5nM, was sufficient to partially inhibit the infectivity of the atypically sensitive IIIB strain of HIV-1 [27], but is several logs less than is required to neutralize primary viruses *in vitro* (typically the µM range; [49]). The two-domain CD4 protein has not been tested for vaginal protection, but a relevant comparison is the differential of ~1000-fold between the concentrations of the b12 MAb that were protective *in vivo* and *in vitro* (Figure 2) [48]. The b12 MAb also targets the CD4 binding site on gp120, although with much greater potency than the two-domain CD4 protein. Right column: The CV-N protein was secreted from engineered lactobacilli to maximal concentrations of 250-400 nM *in vitro*; the biological activity of the secreted CV-N, measured by inhibition of HIV-1 (HXB2-Env pseudovirus) infection of MT4 cells, corresponded to ~20 nM [28]. The CV-N concentrations reported to give complete protection of macaques [34,35] are thus > 4 logs higher than those achieved in lactobacillus cultures.
