# Supplementary material for: Which Topical Microbicides for Blocking HIV-1 Transmission Will Work in the Real World?
Source: PLoS Med. 2006 Aug 15;3(9):e351. doi: 10.1371/journal.pmed.0030351 (PMC1539095; doi:10.1371/journal.pmed.0030351)
Supplement: Supplementary file 4 [file pmed.0030351.sd004.doc]

Text S4. Detailed explanation of figure 3B.

The cartoon illustrates the principal differences between a closed (*in vitro*) and an open (*in vivo*) system for production of an inhibitor by recombinant bacteria. The secreted inhibitory proteins or peptides accumulate in a vigorously aerated bacterial culture during a multi-hour incubation period (i.e., the system is closed; left). For example, the µM concentrations of the gp41-peptides were reached in the late log phase [29] which would correspond to a bacterial density of ~10^9^/ml. In the lumen of the vagina or rectum, the density of recombinant bacteria may be considerably lower, in that the bacteria form only a thin film on the mucosal surface, particularly in the vagina [29,50]. In the murine gut colonized by recombinant *E.coli,* densities of 10^7^ to 10^8^ colony-forming units/g were sustained over 12 days under conditions of continuous selection pressure from an antibiotic [29]. Dilution, degradation and losses into the ever-draining lumenal fluids are likely to act as counter-balances to the secretion of the inhibitors from the bacteria under *in vivo* conditions (i.e., the system is open, right-hand panel). The half-life of the various inhibitors *in situ* is unknown, but seems more likely to be measured in hours than days. The half-life of proteins introduced exogenously into the mouse vagina has been measured as 3-5 hours, and significantly less (0.7 hours) when the mice were ambulatory [51,52]. Inhibitors that are destroyed or lost will need to be continuously replenished to maintain a steady-state concentration of inhibitors within the vaginal fluid and/or in a thin film coating the mucosal surface. Such concentrations cannot readily be estimated because there is no information on the rates of inhibitor production and loss under *in vivo* conditions. The culture data imply that bacterial densities of ~10^8^-10^9^ per ml might be required in the vagina or rectum for the inhibitors to be produced in amounts similar to those achieved *in vitro*, even if we assume (unrealistically) that there would be no losses over time. In pre-menopausal women the density of lactobacilli recorded in a film on the epithelial surface is highly variable, ranging from less than 10^2^ to above 10^8^ cells per ml [50]. Lactobacilli densities exceeded 10^7^/ml in only 3 out of 20 pre-menopausal women without vaginosis [50]**.** In most women, therefore, the concentrations of inhibitors secreted by the colonizing lactobacilli might not approach those achieved *in vitro,* which are themselves orders of magnitude lower than those that protect macaques when similar or more potent inhibitors are applied topically in a gel. Because of, or despite, the theoretical uncertainties about the potential efficacy of the engineered commensal bacteria concept, it is now essential to generate proof-of-concept protection data in a macaque challenge model(s).

**^1^** The bacterial densities refer to typical values for lactobacilli in pre-menopausal women without vaginosis [50], and in the newly colonized murine gut [29], respectively.
